# A New Weak-Coupling Method with Eccentric Dual Bucking Coils Applied to the PRBS Helicopter TEM System

**DOI:** 10.3390/s22072675

**Published:** 2022-03-30

**Authors:** Zhen Ke, Lihua Liu, Longbin Jiang, Shichu Yan, Yicai Ji, Xiaojun Liu, Guangyou Fang

**Affiliations:** 1 Aerospace Information Research Institute, Chinese Academy of Sciences, Beijing 100094, China; kezhen19@mails.ucas.ac.cn (Z.K.); jianglb@aircas.ac.cn (L.J.); yansc@aircas.ac.cn (S.Y.); ycji@mail.ie.ac.cn (Y.J.); lxjdr@mail.ie.ac.cn (X.L.); gyfang@mail.ie.ac.cn (G.F.); 2 University of Chinese Academy of Sciences, Beijing 100049, China; 3 Key Laboratory of Electromagnetic Radiation and Sensing Technology, Chinese Academy of Sciences, Beijing 100190, China

**Keywords:** HTEM, weak-coupling, PRBS method, residual primary field

## Abstract

In the helicopter transient electromagnetic system (HTEM), weak-coupling coils reduce the mutual inductance of the transmitting and receiving coils, which can protect the data acquisition circuit and improve the signal-to-noise of the system. The PRBS HTEM system is a newly proposed multi-receiver EM measurement system, which can effectively identify the system impulse response of the unknown geological model based on the high-precision synchronous recording signal of the PRBS emitted current and induced voltage. However, the standard PRBS current signal is turned on/off very quickly, easily resulting in signal saturation. Concerning this problem, this paper proposes a new weak-coupling structure named eccentric dual bucking coils for the multi-receiver EM system by analyzing the on/off characteristics of PRBS current and the magnetic field distribution of the transmitter–receiver system. It also verifies the feasibility of the proposed structure by Maxwell software simulation. Furthermore, considering the influence of the residual primary field and other factors, the data preprocessing results of the PRBS method and the traditional square wave method are compared by theoretical analysis and data simulation, and the results show that the earlier-time response data can be obtained by PRBS method under the same simulation conditions. Finally, the reliability of the proposed method is verified by ground experiment.

## 1. Introduction

As an efficient geophysical exploration technology, the helicopter transient electromagnetic (HTEM) method shows great adaptability and reliability in complex terrain, and has been widely used in geophysical exploration, such as groundwater monitoring, mineral exploration, geologic mapping, and environmental surveys [1,2,3]. HTEM systems are principally composed of two parts: the towed loop to emit a periodic electromagnetic pulse, and the receiving coils to measure the induction field which contains a wealth of information on the earth resistivity with a penetration depth of several hundred meters, mainly depending on the surface conductance and equipment performance. In practice, a specific periodic current is injected to the transmitting loop and generates the primary field. Then the earth model produces the corresponding secondary field by the eddy current of the objects. We can obtain the target information after the processing of the recorded magnetic field data (*B* or d*B*/d*t*), including flight attitude correction, data stacking, extraction of response and inversion [4]. However, to obtain as much early-time or high-frequency information as possible, the transmitting current pulse is often switched very quickly, leading to a strong primary field signal mixed in received data of the total field, which may result in signal saturation after passing through the gain circuit [5,6]. Therefore, in order to protect the circuit and achieve an accurate measurement, some measures must be taken to suppress the direct coupling between the transmitting coils and the receiving coils.

An appropriate transmitter–receiver layout is the key to reducing the receiver’s dynamic range and improving the system flexibility [7]. Thus, the receiving sensor is naturally placed in a zero-field position so that the residual primary field has little influence on the receiver. One form is to place the receiving sensor directly in a natural zero-field position without bucking coils, a typical example being the SkyTEM developed by the Hydro Geophysics Group. The receiving coils are placed with a vertical distance of 1.5 m above the corner of the transmitting loop, which is attached to a wooden lattice frame [8]. In addition, some systems adopt an approach called offset-loop configuration, which can greatly reduce the primary field by placing the receiving coils far away from the transmitting loop, such as the HeliTEM system developed by the CGG of France [9].

The other form of primary field suppression is to add an anti-flux coil around the receiving coils, which is a dominant weak-coupling mode for large-loop HTEM systems. The Geotech VTEM system, earlier focusing on late-gate data quality for deep mineral exploration, has indicated that placing the receiver in the center of the transmitting coils shielded by a concentric bucking coil could effectively improve the near-surface imaging [10]. Some other HTEM systems with similar configuration include the Xcite system by New Resolution Geophysics [11], the CAS-HTEM by the Chinese Academy of Sciences [12], and so on. Moreover, the AeroTEM-IV system manufactured by Aeroquest (acquired by Geotech) is the first to introduce the eccentric bucking coil into the HTEM system [13]. It makes use of an eccentric single bucking coil to compensate the primary field, and achieves the miniaturization of field compensation to a certain extent. Analogously, Chen and Liu also realize the design and optimization of the eccentric bucking coil, and then apply it to the helicopter system CHTEM-II [14,15].

**Figure 1:**
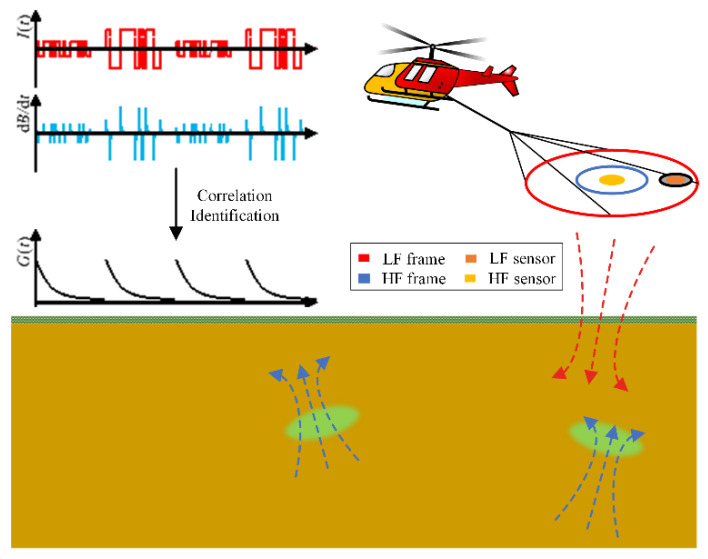
Schematic diagram of the multi-receiver PRBS HTEM system.

In this paper, we design a novel multi-receiver EM system for shallow–deep detection. According to the above analysis, a new weak-coupling structure named eccentric dual bucking coils are firstly proposed and applied to our helicopter TEM system. Compared with the traditional bucking methods, the proposed model can provide a relatively compact sensor structure to meet the space development demand of the multi-receiver EM system. In addition, for improving the SNR and the system performance for all-around object identification, the pseudo-random binary sequence (PRBS), which has characteristics similar to white noise, is firstly used as the transmitting signal for the HTEM system. The PRBS is potentially useful for geophysical exploration because it is also a system identification process where the earth is usually treated as an unknown linear time-invariant system [16]. By using the correlation identification algorithm, we can finally obtain the complex underground resistivity spectrum [17,18]. The PRBS helicopter TEM system (PRBS HTEM) developed by the Aerospace Information Research Institute, Chinese Academy of Sciences, is a newly developed dual-receiver EM system, which can expand the geological conductivity range and increase the scope of its application. Figure 1 shows the main schematic of such HTEM systems with two receiving sensors.

## 2. PRBS Helicopter TEM System

There are two main components in the whole PRBS HTEM system: low-frequency (LF) and high-frequency (HF) subsystems. They use two physically isolated transmitting frames with different configurations to inject a PRBS current with different frequency bands, and work by turns to realize alternating shallow–deep detection. A detailed introduction is given in this section.

### 2.1. Transmitter–Receiver Configuration

**Figure 2:**
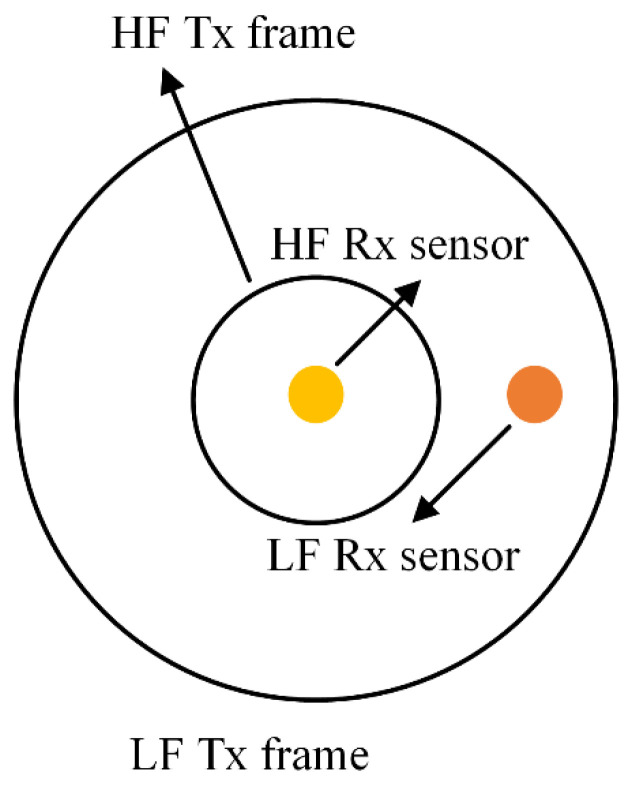
Overall layout of the transmitter–receiver coils.

In the PRBS helicopter TEM system, a bird platform towed to the helicopter by a main cable carries a multi-frequency PRBS transmitter source, LF and HF transmitting coils, and LF and HF receiving coils. Figure 2 shows the coil layout of the PRBS helicopter TEM system. The diameter of the LF and HF frames is 30 m and 15 m, respectively, and they are concentric and coplanar. In order to avoid crosstalk between sensors, the LF and HF sensor coils are placed in the center and away from the center of the bird as designed, respectively, and keep enough distance. All electromagnetic equipment, such as the power transmitter and multi-channel receiver are installed in the cable of the helicopter, while the auxiliary sensors, such as the altimeter, GPS and INS are fixed on the bird platform to provide a stable flight structure.

In order to achieve multifunctional joint detection for shallow and deep targets, the system adopts the time-sharing mode realized by a multi-frequency PRBS pulse source, where the LF and HF sensors receive the secondary field of the LF and HF PRBS signal, respectively. The LF and HF receiving sensors are multiturn coils with an area of 115 m^2^ and 2 m^2^ for measuring the vertical component, and their cut-off frequency is 60 kHz and 220 kHz, respectively. Detailed system specifications are listed in Table 1.

### 2.2. Transmitter Circuit

**Figure 3:**
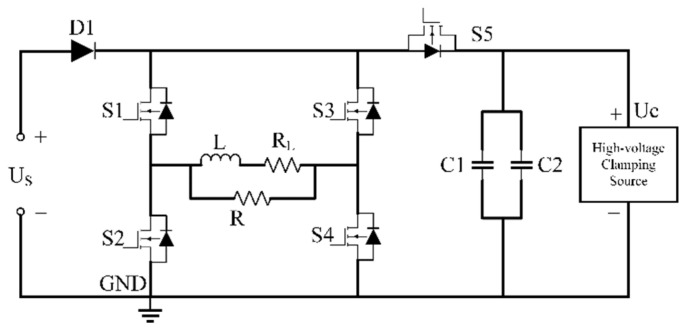
Circuit schematic of PRBS pulse transmitter.

Figure 3 is the designed PRBS transmitter circuit which can provide a PRBS current pulse with a stable frequency. The source Us provides DC power to the transmitting system. S1–S5 are high-power MOSFET modules. The active clamping circuit, composed of a power switch S5 and high-voltage clamping source Uc, provides a stable high voltage to the inductive coil, making the coil current rise and fall, linearly and quickly. The D1 is a fast recovery diode which connects the DC source and full bridge circuit. The full bridge and the clamping circuit is the key part to produce the PRBS waveform by controlling the on/off states of switch S1–S4. The FPGA controlling unit of the transmitter generates a sequential logic signal to control the MOSFET power switches.

### 2.3. Waveform Analysis

In practice, it is difficult for the current waveform to rise and fall ideally, because of the limitations of the switcher module and the inductance of the transmitting loop. To further illustrate the current edge characteristics, the waveform is simulated based on the circuit in Figure 3 using the Matlab Simulink tool.

**Figure 4:**
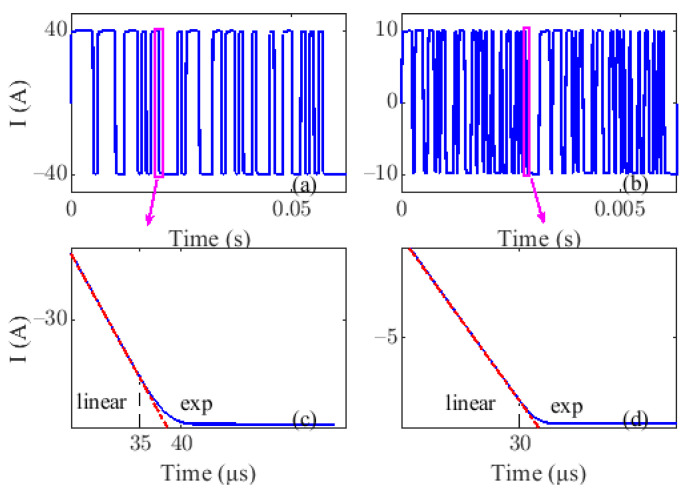
The simulated LF and HF PRBS waveform and its localized current edges. (**a**) PRBS waveform with a frequency of 1 kHz. (**b**) PRBS waveform with a frequency of 20 kHz. (**c**) the enlarged view of LF current edge. (**d**) the enlarged view of HF current edge.

The load coil inductance and resistance are assumed to be 0.5 mH and 1 Ω, referring to the actual measuring results of the transmitting loop. The adjustable clamping voltage is set to around 1000 V. Figure 4a and Figure 4b show the simulated 6-order PRBS waveform with a frequency of 1 kHz (LF) and 20 kHz (HF), respectively. To clearly show current edges, Figure 4c and Figure 4d redraw the localized current of Figure 4a and Figure 4b, respectively. It shows that the LF/HF PRBS current is switched on or off linearly at an early stage, and eventually decays exponentially to zero under the action of the constant voltage clamping circuit and damping circuit. The ramp time of the LF current is close to 40 μs, which will generate a greater primary field at the LF receiving area. The proposed eccentric dual bucking model is applied to solve the problem, and emphatically introduced in the next paper.

## 3. Designment and Optimization of Eccentric Dual Bucking Modeling

This section will introduce the design optimization method of the proposed bucking model used for the LF subsystem in detail.

### 3.1. Calculation of Electromagnetic Field

**Figure 5:**
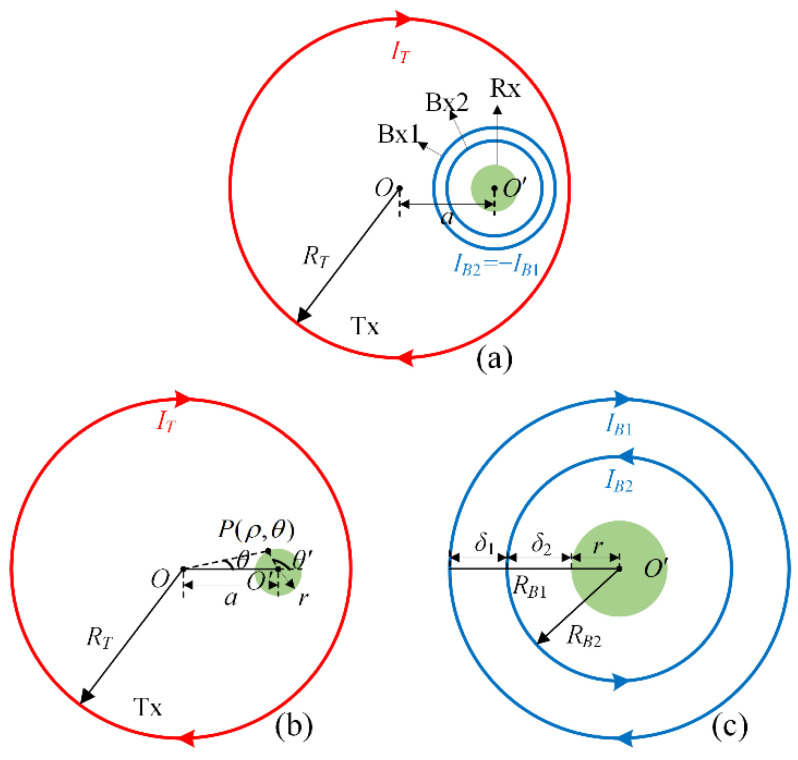
Design principle of eccentric dual bucking model. (**a**) the overall spatial distribution of each submodule in this model. (**b**) the magnetic flux at the receiving area of the transmitting coils. (**c**) the magnetic flux at the receiving area of the bucking coils.

Figure 5a shows the overall distribution of the proposed bucking model. The Tx loop will be taken as a 30-m diameter coil with two turns. The bucking coils at the eccentric position includes two parts with different radii, an outer bucking coil Bx1 and an inner bucking coil Bx2, respectively. The current in Bx1 and Bx2 has the same amplitude and opposite directions, and the current in all coils is of the same amplitude, that is I_T_ = I_B1_ = −I_B2_. Finally, the coil sensor Rx is fixed on the center of the bucking coils by a hard structure, and its radius is 0.6 m. According to the superposition principle of fields, the primary field produced by the bucking coils cancels that produced by the transmitting coils.

As shown in Figure 5b,c, the sensing area is located on the eccentric position of the transmitting coils and the center position of the bucking coils, respectively. Because all coils are coplanar in this transmitter–receiver system, we only consider the z component of the primary magnetic field. In the coordinate system shown in (b), the z-component field of the circular coil with radius R, injected current I, and number of turns N is calculated as follows [19]:

(1) $$B_{P}=\frac{\mu_{0}NI}{4\pi }\left(\frac{2}{R+\rho }K\left(k\right)+\frac{2}{R-\rho }E\left(k\right)\right)$$

where ρ is the radial distance from the observation point P to the origin O, K and E are the first and second kind of complete elliptic integral function, respectively. The term k meets as follows

(2) $$k=\frac{2\sqrt{\rho R}}{\rho +R}$$

According to the complete elliptic integral substitution formula expressed as

(3) $$\{\begin{array}{c} K\left(\frac{2\sqrt{\alpha }}{1+\alpha }\right)=\left(1+\alpha \right)K\left(\alpha \right)\\ E\left(\frac{2\sqrt{\alpha }}{1+\alpha }\right)=\frac{2E\left(\alpha \right)-\left(1-\alpha^{2}\right)K\left(\alpha \right)}{\left(1+\alpha \right)} \end{array}$$

Then (1) can be rewritten as

(4) $$B_{P}=\frac{\mu_{0}NI}{4\pi }\cdot \frac{4RE\left(\frac{\rho }{R}\right)}{R^{2}-\rho^{2}}$$

In the above formula, the complete elliptic integral function E can be calculated as follows

(5) $$E\left(k\right)=\int_{0}^{\pi /2}\sqrt{1-k^{2}\sin^{2}\gamma }d\gamma$$

Combining (4) and (5), the primary magnetic field in the receiving area generated by the transmitting coils can be derived as follows

(6) $$\phi_{T}=\int_{S_{r}}B_{P}ds^{\prime }=\frac{\mu_{0}NI}{4\pi }\cdot \int_{0}^{r}\int_{0}^{2\pi }\int_{0}^{2\pi }f(\rho^{\prime },\gamma ,\theta^{\prime })\rho^{\prime }d\gamma d\rho^{\prime }d\theta^{\prime }$$

(7) $$f=\frac{\sqrt{R^{2}-\left(a^{2}+{\rho^{\prime }}^{2}-2a\rho^{\prime }\cos\theta^{\prime }\right)\sin^{2}\gamma }}{R^{2}-\left(a^{2}+{\rho^{\prime }}^{2}-2a\rho^{\prime }\cos\theta^{\prime }\right)}$$

where a is the offset from O to O′, ρ′ and θ′ represent the integral radius and angle of the transformed coordinates system centered at the origin O′, respectively. Similarly, we can obtain the flux ϕ_B1_ and ϕ_B2_ caused by the bucking coil Bx1 and Bx2 using Equations (6) and (7), respectively. Consequently, the total magnetic flux ϕ_on_ at the receiving coil after compensation and area normalization is calculated as follows

(8) $$\phi_{on}=(\phi_{T}+\phi_{B1}-\phi_{B2})/\pi r^{2}$$

Next, the induced voltage V_on_

(9) $$V_{on}=-n\pi r^{2}G\frac{d\phi }{dt}=-S_{e}\frac{\phi_{on}}{\Delta t}$$

where n represents the number of turns of the receiving coils, G is the amplifier gain of the receiving coils, and S_e_ represents the effective area, generally in the range of 1000 to 10,000 m^2^. Considering the supersaturation problem results from the excessive primary field, the induced voltage V_on_ should not exceed the protection voltage of the data acquisition circuit that is set ±10 V in this system.

In order to ensure that the system has the best allowable error, the residual primary field ϕ_on_ must be as close to zero as possible. According to (8), the relationship between offset a and coil radii R_B1_ (R_B2_) is determined, and then the optimal design of system parameters is realized.

### 3.2. Determination of Bucking Parameters

**Figure 6:**
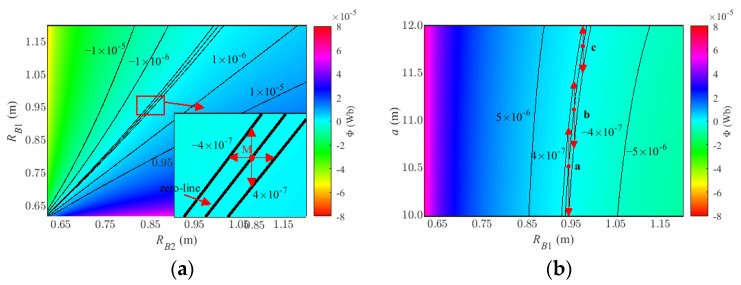
Field calculation results: (**a**) the influence of the inter bucking coil radius R_B2_ and the outer bucking coil radius R_B1_. (**b**) the influence of the receiving coil position offset a and outer bucking coil size radius R_B1_.

According to (9), the received voltage V_on_ depends largely on the residual magnetic flux ϕ_on_. Assuming that the effective area is 1000 m^2^, the term ϕ_on_ must be lower than 4 × 10^−7^ Wb to prevent primary field saturation. Considering the overall platform structure, the bucking coils are installed approximately 10–12 m from the transmitting center. Expectantly, an ideal zero-level line can be calculated; the coordinates of the points on the line are the compensation parameters we need. The residual magnetic flux distribution diagram when a is equal to 11 m is shown in Figure 6a; the middle contour is the zero-level line. From the picture, the smaller the bucking radius is, the more intensive the contour lines of the residual magnetic flux, which means that the model is more sensitive to coil size error. Because of the thermal expansion and cold contraction effect, and metal wire machining error, the bucking coil size should avoid being too small. Therefore, the inner bucking coil R_B2_ can be selectively confirmed to be 0.850 m when the allowable size error of the coil is 5 mm. The position of the compensation point is enlarged and shown in the lower right corner of Figure 6a, the red arrow means the allowable size error from zero-level to the limit value.

Next, to determine a locally optimal offset value a, the effect of the receiving coils position offset and outer bucking coil size is illustrated in Figure 6b. The bucking coil radius ranges from 0.6 m to 1.2 m, and the offset value ranges from 10 m to 12 m. The middle contour is the zero-level line, and the respective contours for ±4 × 10^−7^ Wb are also plotted. The letters a–c represent selected three representative compensation points at different offsets and the corresponding length of the red arrow represents the maximum allowable horizontal offset error ΔH. Table 2 lists compensation parameters of these points and their allowable horizontal deviation ΔH. As shown in the table, ΔH is inversely proportional to the offset a, which indicates that reducing the offset is beneficial to improving the fault tolerance of the model. Finally, the offset a is set to 10.5 m, and the corresponding inner and outer bucking coil radius is 0.850 m and 0.943 m in this paper, respectively.

## 4. Simulation Results

To further evaluate the performance of the proposed method, the simulation test is described in this section.

### 4.1. Simulation of Bucking Model

Maxwell is a high-performance EM analysis software and used to simulate the eccentric dual bucking coils model. The simulation parameters are listed in Table 3. The geological model is set to a uniform half space, and the coil model is located 30 m above the ground.

**Figure 7:**
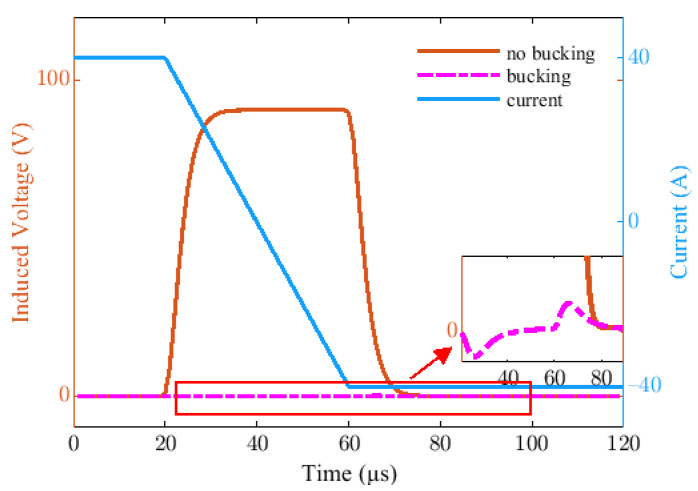
The received induced signals when the primary field signal is compensated or not compensated.

A step pulse wave is used to replace the ideal cut-off edge, the received induced signal when the primary field signal is compensated or not compensated are given in Figure 7. Apparently, if any measures are not taken, the amplitude of the received signal is too large to saturate and damage the back-end circuit. While the eccentric dual bucking coils model is used, the primary field coupling is suppressed to nearly 0, and the amplitude of the secondary field is approximately 100 mV. The simulation results verify the effectiveness of the proposed bucking model.

### 4.2. Quantitatively Analysis of the Difference betweeen PRBS Method and Traditional Method

In a traditional TEM method, the square wave, triangle wave, half cosine wave and other waveforms are generally treated as the transmitted waveform, and we obtain the secondary field of the earth system by extracting the received EM data during the current shutdown. However, system bandwidth and residual primary field greatly affect the ability of the system to obtain an early response. Next, quantitative analysis of the influence of these factors on the PRBS method and the traditional method is given.

A typical input–output mathematic model of the TEM system is introduced in Appendix A. For the PRBS method, we can directly obtain the unknown system response G_p_(t) with a time-domain correlation identification algorithm as follows

(10) $$G_{p}(t)=Kh(t)+g_{2}(t)*h(t)$$

where K is a constant used for measuring the size of the residual primary field. However, for the traditional method, we use the full-wave response of the current waveform i(t) as follows

(11) $$G_{t}(t)=i^{\prime }(t)*G_{p}(t)$$

Taking the step pulse waveform with a ramp time of Δt as an example, the above formula can be rewritten as follows

(12) $$G_{t}(t)=\frac{1}{\Delta t}\int_{0}^{\Delta t}\left(u(\tau )-u(\tau -\Delta t)\right)\cdot G_{p}(t-\tau )d\tau$$

where u(t) is the unit Heaviside function. In order to obtain the analytical solution of (10) and (12), we use an exponential model and equivalent circuit model to represent g_2_(t) and h(t), respectively [20,21,22], their transfer functions shape like

(13) $$G_{2}(s)=\frac{A\tau }{\tau s+1}$$

(14) $$H(s)=\frac{1}{1+2\zeta \frac{s}{w_{p}}+\frac{s^{2}}{w_{p}{}^{2}}}$$

where A and τ are the amplitude and time constant of an exponential function, respectively, and ζ and w_p_ are the damping coefficient and resonant frequency of the 2-order sensor system, respectively.

#### 4.2.1. Different Resonant Frequency w_p_ and Damping Coefficient ζ

**Figure 8:**
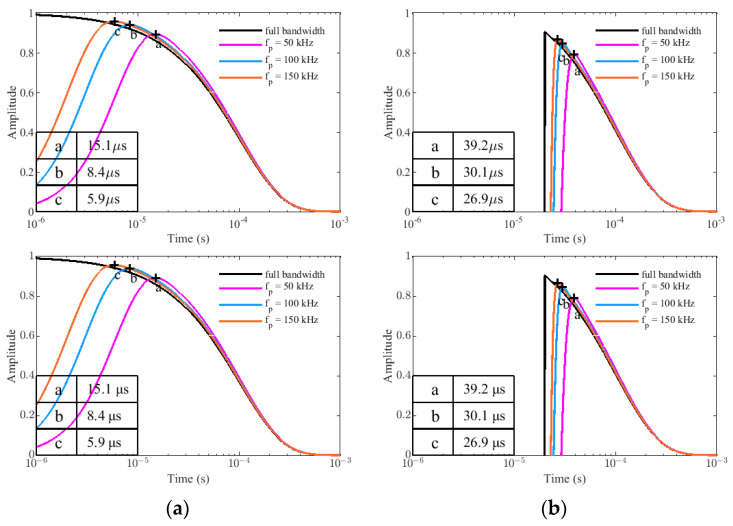
The simulation results in different resonant frequencies w_p_: (**a**) the target response of PRBS method G_p_(t). (**b**) the target response of traditional method G_t_(t).

Assume that A = 1, τ = 1 × 10^−4^ s, K = 0, ζ = 0.9, and Δt = 20 × 10^−6^ s, the simulation results of G_p_(t) and G_t_(t) in full bandwidth, f_p_ = 50 kHz, f_p_ = 100 kHz, and f_p_ = 150 kHz are shown in Figure 8, where the full bandwidth means H(s) = 1. The peak times of different resonant frequencies are marked with the symbols a, b, and c in the figure, and their values are given in the lower left corner of the picture. From the picture, increasing the resonant frequency of the receiving coils can obviously decrease the peak time of the curve and reduce the response error, which means that we can obtain earlier time-gate data. Comparing (a) and (b), the peak-times of the target response of the PRBS method in f_p_ = 50 kHz, f_p_ = 100 kHz, and f_p_ = 150 kHz are 24.1 μs, 21.7 μs, and 21.0 μs earlier, respectively, because it removes the influence of the transmitting waveform.

**Figure 9:**
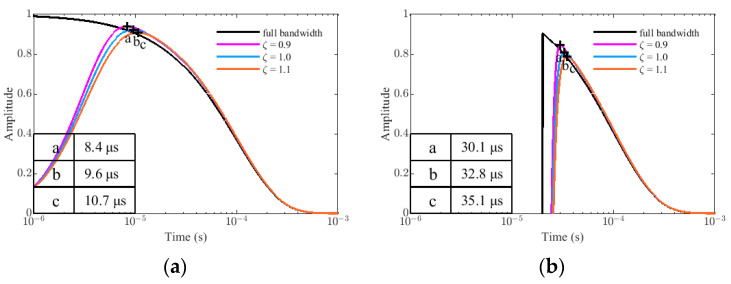
The simulation results in different damping coefficients ζ: (**a**) the target response of PRBS method G_p_(t). (**b**) the target response of traditional method G_t_(t).

Under the same simulation conditions, assume that f_p_ = 100 kHz, the simulation results of G_p_(t) and G_t_(t) in full bandwidth, ζ = 0.9, ζ = 1.0, and ζ = 1.1 are showed in Figure 9. The peak times of different damping coefficients are also marked with the symbols a, b, and c in the figure, and their values are given in the lower left corner of the picture. Apparently, reducing the damping coefficients can also shorten the peak time of the curve because the damping coefficient and the resonant frequency collectively determine the bandwidth of the sensor system. In practical applications, we need to adjust the matching circuit to change the sensor parameters to meet the requirements of different measurement targets (different τ).

#### 4.2.2. Different Primary Field Coefficient K

In actual geological surveys, it is difficult to fully compensate the mutual inductance between transmitting coils and receiving coils, because of the unequal transmitting current or unstable system state. In order to illustrate the influence of primary field interference on the PRBS method and the traditional method, the target response of the different primary field coefficient K is analyzed in this part.

**Figure 10:**
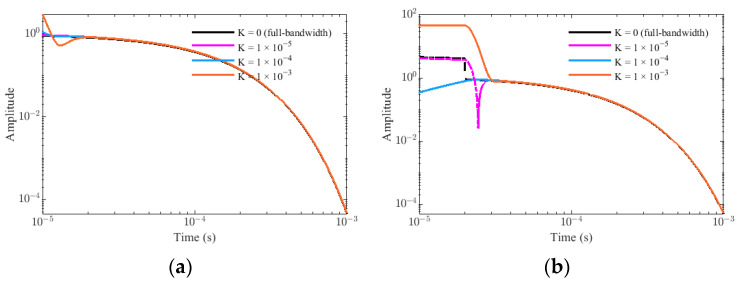
The simulation results in different residual primary field K: (**a**) the target response of PRBS method G_p_(t). (**b**) the target response of traditional method G_t_(t).

**Figure 11:**
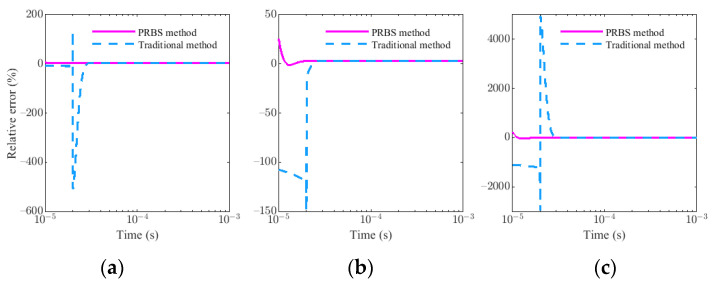
The comparison of response relative errors between PRBS method and traditional method in different primary field coefficient K: (**a**) K = 1 × 10^−5^. (**b**) K = 1 × 10^−4^. (**c**) K = 1 × 10^−3^.

Similarly, assume that A = 1, τ = 1 × 10^−4^ s, f_p_ = 100 kHz, ζ = 0.9, and Δt = 20 × 10^−6^ s, the simulation results of G_p_(t) and G_t_(t) in K = 0, K = 1 × 10^−5^, K = 1 × 10^−4^, and K = 1 × 10^−3^ are shown in Figure 10. It is found that the influence of the residual primary field on the target response is mainly concentrated in the early stages, and increases with the increase in the parameter K, but the influence in the middle and late stages is negligible. The comparison of response relative errors between the PRBS method and the traditional method with different K is showed in Figure 11. As we can see, the error caused by the residual primary field for the PRBS method is mainly concentrated near t = 0 and reduces rapidly because the unit impulse response of the sensor system h(t) behaves as a rapidly decaying sin or sinh function. If the waveform is a step pulse with the ramp time Δt, the influence is mainly concentrated near t = Δt and lasts for some time because of the transition effect of the sensor. Therefore, we can obtain the earlier response data by PRBS method even if there is residual primary field.

### 4.3. Data Sysnthesis and Preprocessing of PRBS HTEM System

**Figure 12:**
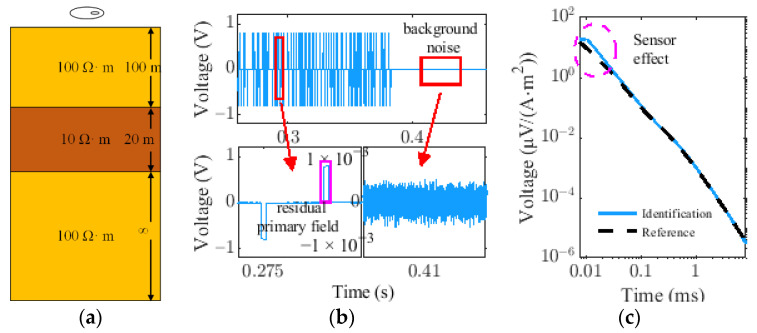
(**a**) Layered earth model. (**b**) 1-D Synthetic EM data which contains residual primary field and gaussian noise. (**c**) Comparison of the reference impulse response and identified model impulse response obtained by correlation identification method.

Next, we adopt the model as shown in Figure 12 to synthesize the TEM data. The radius of the transmitting coil is 15 m, the number of turns is two, the transmitting current is 40 A, the receiving area is 115 m^2^, and the system bandwidth is 60 kHz. In the TEM forward modeling, a quadrature-with-extrapolation (QWE) numerical algorithm is used for the impulse response calculation of the eccentric position of the circular loop [23]. As shown in Figure 12a, a three-layer earth model with a background resistivity of 100 Ω·m is designed, and there is a 20-m-thick and low-resistivity layer at a depth of 100 m in the model with a resistivity of 10 Ω·m. The transmitted current signal is simulated based on the Matlab Simulink described in Section II, and the corresponding 1-D synthetic data are given in Figure 12b. Moreover, the Gaussian noise and residual primary field are added to the synthetic data to simulate the real electromagnetic environment, and the lower part of Figure 12b magnifies a part of the full-wave EM response and background noise.

**Figure 13:**
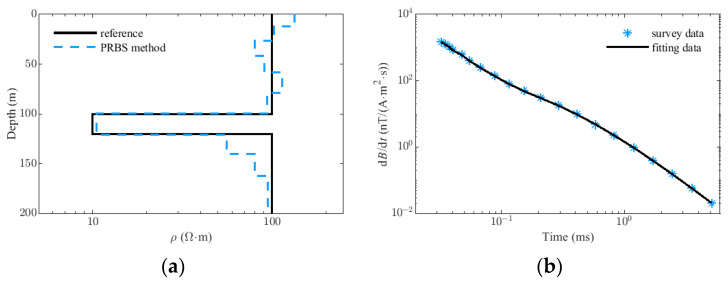
The inverted model based on the survey data of the PRBS method. (**a**) Reference earth model and inversion model. (**b**) Response data and its inversion fitting data.

The time-domain correlation identification algorithm is used to extract the system impulse response of the earth model from the PRBS EM data, and its detailed introduction is given in Appendix A. Figure 12c is the comparison of the identified model impulse response from the synthetic data under the ideal simulation conditions and the theoretical model response. The black curve represents the theoretical response of the geological model calculated by the QWE numerical algorithm, and the blue curve represents the extracted impulse response from the PRBS EM data. In the early stage, due to the limitation of system bandwidth, the identified response curve has an obvious filtering effect (60-kHz lowpass filter has been applied), and in the middle and late stage, it is in good agreement with the theoretical model, which verifies the validity of the time domain correlation identification method. Inverting these data sets obtained by PRBS methods, the inversion model (20 channels) is given in Figure 13a. The results illustrate that the inversion model of the PRBS survey data is in good agreement with the reference model. As is shown in Figure 13b, data fit is near perfect and gives no indication of an erroneous model.

**Figure 14:**
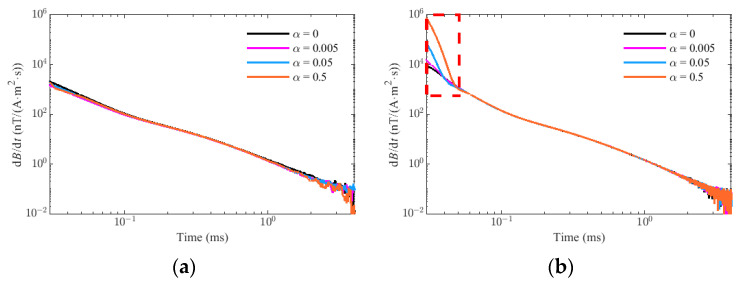
The comparison of the preprocessing results between square wave method and PRBS method under different residual primary field conditions. (**a**) PRBS method (**b**) square wave method.

Figure 14 shows the comparison of the preprocessing results between the square wave method and the PRBS method under different residual primary field conditions, where they have the same current amplitude and the square wave is bipolar with the ramp time of 20 μs. The parameter α used to measure the residual primary field is defined as the relative ratio of the remaining primary field to the complete primary field. When the normalized noise amplitude is 0.1 nT/(A·m^2^·s) and a 60-kHz lowpass filter is applied, the preprocessing results of the PRBS method and the square wave method in different parameters α is given in Figure 14a,b, respectively. From the picture, the residual primary field will distort the early response after the turn-off time for the square wave method, resulting from the filtering effect of the sensor on the residual primary field. Moreover, the larger the parameter α is, the more serious the distortion of the early response will be, which will make it difficult to fit the data for the earlier gates, as well as the loss of potentially valuable near-surface information. However, the essence of the PRBS method is to obtain the system impulse response of the geological model, and it can resist primary field interference to some extent, according to the results of correlation identification, which is consistent with the results of theoretical analysis.

## 5. Experiment Results

In order to further verify the effectiveness of the proposed bucking model and the PRBS system detection capability (LF module), a ground field test is completed, and the measured field data are processed and analyzed in this section.

**Figure 15:**
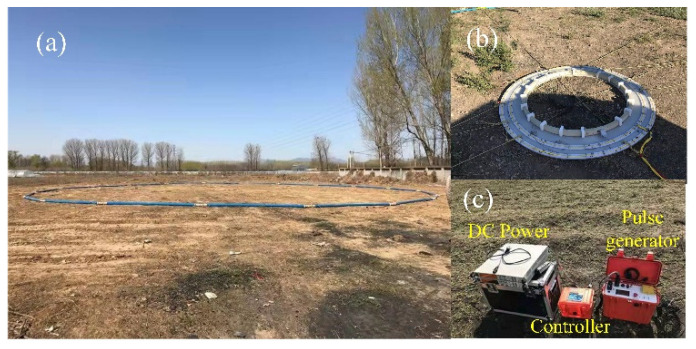
(**a**) The LF circular launching frame. (**b**) The proposed bucking structure and LF sensor. (**c**) The DC power supply and PRBS pulse transmitter.

**Figure 16:**
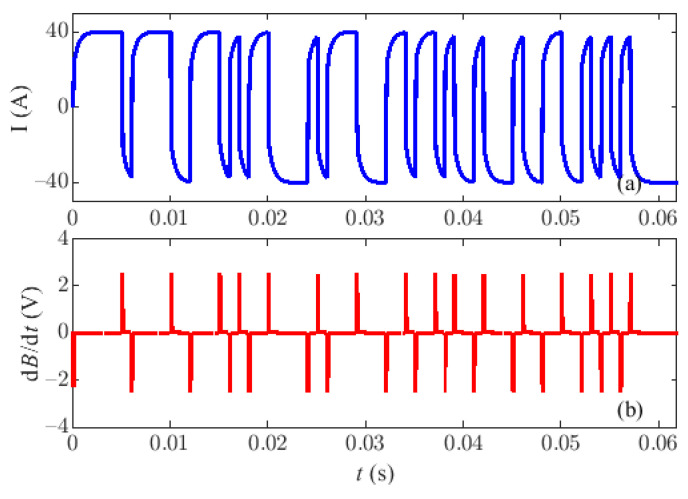
The recorded data. (**a**) The recorded transmitted current signal, (**b**) The recorded induced TEM signal.

A field exploration is implemented at Zhangjiakou City, Hebei Province, China, and the relevant components of the LF PRBS HTEM prototyping system are configured on the ground as shown in Figure 15. The diameter of the LF launching frame is 30 m (two turns), the equivalent receiving area is 115 m^2^. A 2-kHz PRBS wave with an amplitude of 40 A is emitted, and the sampling rate of the receiving system is 192 kHz. The recorded current data and induced voltage data are shown in Figure 16. It is worth noting that the linearity of the emission current during the on/off time decreases, mainly resulting from the difference between the designed and the actual system parameters. During the on-time period, the induced voltage is mainly generated from the residual primary field due to the inadequate primary field compensation. From the picture, the amplitude of the maximum induced voltage is about 2.5 V, which is lower than the saturation voltage and meets the expected demand. Furthermore, it is difficult for the PRBS method to directly obtain the secondary field signal by extracting the turn-off signal, as in the traditional square wave method, because of the difference in the width of the symbols. However, the correlation recognition method in the time domain is an effective tool to obtain the impulse response of an unknown geological system, and the identified signal includes the electrical information that we need about the underground medium.

**Figure 17:**
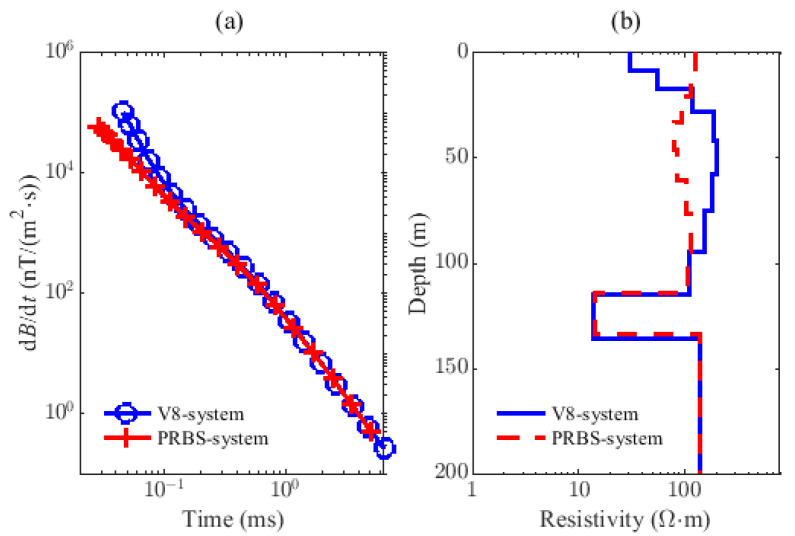
The data preprocessing results of PRBS system and V8 system. (**a**) The preprocessed observed data, (**b**) The inverted resistivity model of sounding shown in (**a**).

In order to verify the effectiveness of the proposed method, we conduct a set of ground experiments using the PRBS system and the V8 system, respectively, at the same observation site, and it is also known that there is an aquifer in the observation point about 120 m underground. The V8 system emits continuous square waves with a frequency of 25 Hz, and the ramp time of the current is around 19.6 μs. The current amplitude of both is 10 A. The data processing results of the two systems are shown in Figure 17. Figure 17a shows the induced voltage data after preprocessing (20 channels), and the corresponding inverted resistivity models (10 layers) are shown in (b). From the figure, the attenuation curve of the PRBS system is in good agreement with that of the V8 system in the late stage, and a low resistivity layer with nearly 10 Ω·m appears 120 m underground, which is a very good overall match to priori knowledge. Experimental results confirm the reliability of the LF PRBS system.

## 6. Conclusions

In this paper, a new eccentric dual bucking coils model is proposed for the PRBS HTEM system. The system contains LF and HF, two subsystems to realize a shallow–deep–shallow alternate detection mode. The Simulink simulation tool is used to simulate the PRBS current waveform of the inductive load circuit in two working modes, and the on/off characteristics of the current are analyzed. Next, the mathematical expressions of the magnetic field for the eccentric position of a circular coil are presented, and the design of the proposed bucking model is realized according to the ramp time of the simulation. In fact, this model can provide an extremely compact sensor structure which is beneficial to the layout of the multi-receiver system, but it is bound to sacrifice a relatively stable electromagnetic environment due to the off-center location. In Section 4, the designed bucking model is simulated by Maxwell software, and the results show that it can effectively reduce the primary field, with the induced voltage signal shrunk by 1000 times. Furthermore, quantitative analysis of the target response function of the PRBS method and the traditional method under the conditions of different bandwidth and residual primary field is realized. It is found that the PRBS method does better in the case of primary field disturbance and can obtain earlier-time response data beneficial to reflect potentially valuable near-surface information, and the correlation identification algorithm is verified by using PRBS synthesizing EM data. Finally, a ground field exploration is implemented for the LF PRBS system, and it shows that the prototype testing system can effectively reduce the primary field and achieve the desired target detection effect. Experimental results confirm the reliability of the proposed method. Future work is further completing the flight experiment of the whole PRBS helicopter TEM system.

## Figures and Tables

**Table 1:** System parameters for the LF and HF modes.

Items	Low Frequency	High Frequency
Transmitter area	1400 m^2^	700 m^2^
Peak current	40 A	10 A
Peak moment	56 kA·m^2^	7 kA·m^2^
Code frequency	1–20 kHz	20–50 kHz
Order	6–8	8–10
Sample interval	5 μs	1 μs

**Table 2:** Selected representative points at different offsets (R_B2_ = 0.850 m).

Points	a (m)	R_B1_ (m)	ΔH (m)
a	10.50	0.943	0.93
b	11.10	0.955	0.71
c	11.77	0.975	0.49

**Table 3:** Simulation parameters of bucking model.

Model Parameters	Value
Tx waveform	Step pulse
Ramp time	40 μs
Tx current	40 A
Tx coils radius	15 m
Rx coils radius	0.6 m
Bucking coils radius	0.850 m/0.943 m
Terrain clearance	30 m
Earth resistivity	100 Ω·m
Sample interval	1 μs

## Appendix A. Time Domain Correlation Identification Algorithm

The input-output model between the received signal u(t) received by the coil sensor and the emission current signal i(t) can be built as follow

(A1) $$u(t)=u_{1}(t)+u_{2}(t)+n(t)=-\frac{d}{dt}i(t)*(\frac{\alpha \phi_{T}}{I_{m}}\delta (t)+g_{2}(t))*h(t)+n(t)$$

where the right-hand equation contains the residual primary field u_1_(t), secondary field u_2_(t) and gauss noise n(t). The item α is the residual primary field parameter, ϕ_T_/I_m_ represents the mutual inductance of the transmitting coils and the receiving coils, δ(t) represents Dirac delta function, g_2_(t) represents the unit impulse response of the geophysical model, h(t) represents the unit impulse response of receiving sensor, and n(t) represents the gauss noise. Correlation operation is carried out with the input signal i(t) on both sides of the above equation:

(A2) $$i(t)⊗u(t)=-i(t)⊗i(t)*\frac{d}{dt}(\frac{\alpha \phi_{T}}{I_{m}}\delta (t)+g_{2}(t))*h(t)+i(t)⊗n(t)$$

where the symbol ⊗ represents the periodic correlation operation, which can be done quickly by fast Fourier transform. The term i(t) ⊗ n(t) can be ignored because of the independence of the PRBS signal and the gauss noise. Consequently, the formula (A2) can be rewritten by using the symbol R_ui_(t) and R_ii_(t) to represent the cross-correlation and auto-correlation as follows:

(A3) $$R_{iu}(t)\approx -R_{ii}(t)*G^{\prime }(t)$$

(A4) $$G(t)=\frac{\alpha \phi_{T}}{I_{m}}h(t)+g_{2}(t)*h(t)$$

After discretization, the convolution above can be derived as

(A5) $$R_{iu}(k)\approx -\sum_{j=0}^{N}R_{ii}(k-j)G^{\prime }(j)\Delta$$

where N and Δ represent the number of sampling points over a duration and sampling interval, respectively. Next, (A5) can be expressed in matrix form as follow

(A6) $$R_{iu}\approx R_{ii}G^{\prime }$$

(A7) $$R_{ii}=\left[\begin{array}{cccc} R_{ii}\left(n_{2}\right) & R_{ii}\left(n_{2}-1\right) & \cdots & R_{ii}\left(n_{2}-N+1\right)\\ R_{ii}\left(n_{2}+1\right) & R_{ii}\left(n_{2}\right) & \cdots & R_{ii}\left(n_{2}-N+2\right)\\ \vdots & \vdots & \ddots & \vdots \\ R_{ii}\left(n_{2}+N-1\right) & R_{ii}\left(n_{2}+N-2\right) & \cdots & R_{ii}\left(n_{2}\right) \end{array}\right]$$

(A8) $$R_{iu}=\left[\begin{array}{c} R_{iu}\left(n_{1}\right)\\ R_{iu}\left(n_{1}+1\right)\\ \vdots \\ R_{iu}\left(n_{1}+N-1\right) \end{array}\right]$$

where n_1_ and n_2_ are the peak coordinates of cross-correlation function R_iu_ and auto-correlation function R_ii_, respectively. Then, the optimally estimated ${\hat{G}}^{\prime }$ can be obtained as follow

(A9) $${\hat{G}}^{\prime }={\left(R_{ii}{}^{T}R_{ii}\right)}^{-1}R_{ii}{}^{T}R_{ui}$$

Finally, the estimated earth secondary response ${\hat{G}}^{\prime }$ can be obtained by the integral operation and amplitude correction based on its differential form ${\hat{G}}^{\prime }$.
